# Communicating about Data to Achieve Change

**DOI:** 10.34133/2022/9821697

**Published:** 2022-08-30

**Authors:** Kevin D. Frick

**Affiliations:** ^1^Johns Hopkins Carey Business School, USA; ^2^ Johns Hopkins Bloomberg School of Public Health (Departments of Health Policy and Management and International Health) USA; ^3^ Johns Hopkins School of Medicine (Department of Ophthalmology) USA

Even with plans for collecting, managing, and analyzing high-quality data, drawing meaningful conclusions, and writing an exceptional peer-reviewed journal article, there is no guarantee that the findings will be translated into change in the organization, delivery, or financing of health care that will result in better health outcomes for a target population or society at large. The process of turning well-collected data with high-quality analyses and well-drawn conclusions into changes in health care requires clear and careful communication. The importance of timely and clear communication continues to grow as datasets become more complex, research teams become more interdisciplinary, and the speed demanded by business and public health decision-making seems to grow faster by the day. Researchers need to know how to communicate with other researchers with expertise in different fields and with the general public. Successful communication requires action from the researcher and is intended to inspire action in stakeholders who can help bring about change. With action as both an input and an output in the change process, I have developed and utilized a model with *ACTION* as the acronym. 

*ACTION* includes knowing with whom to *associate* and how to associate with them, building *connections* that are necessary for moving to action, *telling* people about the changes that are recommended based on the analysis of the data collected, *inspiring* action, keeping people *oriented* about the process of change, and *nourishing* the relationships so that others continue on the journey to change. Elaboration on each of these steps is important. While elaborating, I will share my experience using *ACTION* related to eye care and blindness. Many members of my family have worn glasses, and the number of individuals globally for whom the only barrier to visual function is a pair of glasses is surprisingly large at 2.29 million [1]. My work has extended over many years as this is a subset of my research and research is only a part of my job. During this time, governments and nongovernmental organizations have dedicated more resources to preventing the need for glasses and providing access to glasses over time; the World Health Assembly launched a plan for universal eye health [1], and I served on a nonprofit board, gave a congressional briefing, and am organizing a webinar on social determinants of eye health disparities. 

## 1. Associate

For *associating* with other relevant stakeholders, finding opportunities begins with any chance to network or connect; after an initial meeting, associating can move to pitching an idea based on the findings from high-quality data collected and analyzed. Opportunities occur in the workplace, at conferences, and at networking events. The first meeting may require only moments of introduction, but making the pitch will require stakeholders’ (hopefully, undivided) attention to listen to a story about the data, the findings, and an emotional anecdote to bring the story to life. The strength of interpersonal associations depends on first impressions, which are related to demonstrated warmth and competence [2].

Most researchers are likely to be perceived as having a high level of competence in their field—they generate hypotheses, manage data, perform analyses, write up their interpretations of results, etc. Some researchers may benefit from developing a capacity to project warmth to provide audiences and collaborators with a positive first impression and to prepare audiences and collaborators to receive the information a researcher wants to share about their findings. I have worn glasses since 1981 and have wanted to make a difference in access to services that impact people’s ability to see. When I was first introduced to one of the world’s leading ophthalmic epidemiologists more than two decades ago, I made sure to present myself as knowing how to and being able to execute steps to conduct cost-effectiveness research and to come across as a colleague interested in more than my own work and interested in strong collaboration.

## 2. Connection

Once an association is initiated, continuing development of the *connection* with each relevant stakeholder is critical. The stakeholders with whom a researcher should connect may include those who control financial resources, those who need care, those who make decisions about care, and others (like family) affected by the care. When networking with relevant stakeholders, the researcher needs to recognize that not all stakeholders will be experts, so communication through telling stories should be broadly understandable.

Over the years, I have developed connections not only with the leading ophthalmic epidemiologist but also with leading optometrists and ophthalmologists, occupational therapy providers and other low-vision specialists, leaders in nonprofit organizations dealing with blindness and eye health, and leaders in relevant industries. My communication skills have needed to span conversations with those with clinical expertise, scientific expertise, and business expertise. Solutions to eye health and vision challenges require interacting with different types of providers and parts of industry and nongovernmental organizations.

## 3. Telling

Making a story broadly understandable to a wide variety of stakeholders requires avoiding acronyms, using as little field-specific terminology as possible, and avoiding sounding like a lecture. The story should be engaging and focused and touch the stakeholders’ emotions as well as their intellect. Researchers should be prepared not only to tell their story once but to tell it multiple times. At times, the same stakeholder will need to be told more than once as a thorough understanding requires the story to be heard on multiple occasions. There are also likely to be many stakeholders who need to hear the story at least once and at different times. The story should be told efficiently with few digressions, as individuals have a finite attention span.

Over the years, I have done two things to focus on storytelling: participating in training regarding storytelling and talking with patients whose stories can enliven the population-level statistics that I analyze and write about.

## 4. Inspiring

To *inspire* stakeholders to take action, the story must be persuasive. A model of persuasion begins with a story or startling statistic to capture attention, continues by pointing to the need for a solution describing the scope of the problem, moves on to describing how the problem will be solved, provides a vision of how the world will be better with the solution or worse without, and concludes by giving the stakeholders a clear action to take [3]. Combining the model of persuasion with a framework for telling stories includes beginning with a clearly described situation, a middle that details an obstacle and action taken, and an ending with results [4] that helps to hold stakeholders’ attention and increase the probability of inspiring action.

This process involves moving beyond the standard peer-reviewed journal articles. I have spoken in radio interviews, have been interviewed for a documentary, have spoken at briefings, and am organizing a webinar. This reflects the range of opportunities to talk about science in combination with stories to inspire people (particularly in industry, not-for-profit organizations, and government) to consider allocating resources to solve problems.

## 5. Oriented

Even with stakeholders inspired to take a first step on the journey of change, change is usually an extended process rather than being instantaneous. Stakeholders need to be kept *oriented* about progress toward change and reminded that perseverance and grit are required to translate findings, conclusions, and recommendations into health care system and societal change. Orientation regarding progress and required perseverance is just one step in the process of ongoing nourishment of the relationships with stakeholders.

For many years, I sat on an advisory board for the Vision Impact Institute. That organization sent updates to stakeholders on a regular basis to keep them apprised of the latest concerns with respect to uncorrected refractive error and the latest attempts to make glasses more accessible.

## 6. Nourishment

Nourishment also requires the researcher who has produced the findings with the data to maintain contact; provide new data; share additional anecdotal, persuasive stories; and create the opportunity for multiple types of engagement. These can include in-person and virtual meetings, one-on-one and group meetings, and one-way and two-way communication mechanisms.

I have nourished relationships at professional meetings, for vision specifically at the meetings of the Association for Research in Vision and Ophthalmology, which attracts a combination of researchers, providers, industry, and not-for-profit organizations. When I attend these meetings, I do not solely present my work, but I use the meetings as a networking opportunity to communicate with a broad array of relevant stakeholders from the time of conceptualizing my research plan to getting my research published to seeking to turn research into changes in the financing, organization, or delivery of care or public health measures.

## 7. Conclusion

The steps in the *ACTION* model do not guarantee that findings will be translated into change; however, they help maximize the probability that the findings from carefully collected, carefully managed, high-quality health and health care data will be used to make substantive and positive changes in the organization, provision, or financing of care.
